# Cohort design to assess the association between post-hospital primary care physician follow-up visits and hospital readmissions

**DOI:** 10.1097/MD.0000000000031830

**Published:** 2022-11-18

**Authors:** Noah Kojima, Marielle Bolano, Andrea Sorensen, Chad Villaflores, Daniel Croymans, Eve M. Glazier, Catherine Sarkisian

**Affiliations:** a David Geffen School of Medicine at University of California Los Angeles, Department of Medicine, Los Angeles, CA, USA; b David Geffen School of Medicine at University of California Los Angeles, Division of General Internal Medicine and Health Services Research, Department of Medicine, Los Angeles, CA, USA; c VA Greater Los Angeles Healthcare System Geriatric Research Education and Clinical Center, Los Angeles, CA, USA.

**Keywords:** post-hospital follow up, post-hospital visit, primary care, readmission

## Abstract

While multifaceted post-hospitalization interventions can succeed in preventing hospital readmissions, many of these interventions are labor-intensive and costly. We hypothesized that a timely post-discharge primary care physician (PCP) visit alone might prevent hospital readmission. We conducted a retrospective cohort study to assess whether post-hospitalization PCP visits within 14 days of discharge were associated with lower rates of 30-day hospital readmission. In a secondary analysis we also assessed: whether visits with a PCP at 7-days post-discharge changed rates of hospital readmissions and whether post-hospitalization PCP visits were associated with decreased 90-day hospital readmissions. We included all adults with a PCP who were discharged from an inpatient medical service in a large, urban integrated academic health system from January 1, 2019 to September 9, 2019 in our analysis. We performed unadjusted bivariate analyses to measure the associations between having a PCP visit within 14 and 7 days of discharge and hospital readmission within 30 and 90 days. Then we constructed multivariate logistic regression models including patient medical and utilization characteristics to estimate the adjusted odds of a patient with a post-hospitalization PCP visit experiencing a 30-day hospital readmission (primary outcome) and 90-day readmission (secondary outcome). A total of 9236 patients were discharged; mean age was 57.9 years and 59.7% were female. Of the study population, 35.6% (n = 3284) and 24.1% (n = 2224) of patients had a post-hospitalization PCP visit within 14 days and or 7 days, respectively. Overall, 1259 (13.6%) and 2153 (23.3%) of discharged patients were readmitted at 30 and 90 days, respectively. In unadjusted analyses, having a post discharge PCP visit was not associated with decreased hospital readmission rates, but after adjusting for sociodemographic, medical and utilization characteristics, having a post-hospitalization PCP visit at 14 and 7 days was associated with lower hospital readmission rates at 30 days: 0.68 (95% CI 0.59–0.79) and 0.76 (95% CI 0.66–0.89), respectively; and 90 days: 0.76 (95% CI 0.68–0.85) and 0.80 (95% CI 0.70–0.91), respectively. In this large integrated urban academic health system, having a post-hospitalization PCP visit within 14- and 7-days of hospital discharge was associated with lower rates of readmission at 30 and 90 days. Further studies should examine whether improving access to PCP visits post hospitalization reduces readmissions rates.

## 1. Introduction

Hospital readmissions are a major source of national healthcare expenditures,^[1]^ are associated with poor outcomes,^[2]^ and use a significant portion of hospital resources.^[3]^ Up to 1 quarter of hospital readmissions are preventable.^[1,4]^ Reducing readmission rates is a high priority goal for healthcare reform at an institutional and national level—with federal policy including the 2012 Patient Protection and Affordable Care Act established by the Centers for Medicare & Medicaid Services’ Hospital Readmissions Reduction Program.

Prior research suggests that in some select settings, timely follow-up visits with primary care physicians (PCP) reduce rates of hospital readmissions. Wiest et al reported that among a retrospective cohort of 1531 adult Medicaid patients in Camden, New Jersey, post-hospital discharge connection to a primary care provider with a 7-Day Pledge program was associated with fewer 30- and 90-day readmissions when compared to patients with less timely primary care follow up.^[5]^ The 7-Day Pledge program is a citywide infrastructure that encompasses 12 primary care practices and engages patients while there are still in the hospital to connect them to a primary care appointment. In contrast, a retrospective analysis conducted by Gurwirtz et al among a cohort of 1870 patients aged 65 years or older in a large multispecialty group practice did not show an association between follow-up PCP visits and fewer readmissions.^[6]^ More labor-intensive interventions in other settings, such as increasing cross-site communication and using transition coaches successfully decreased risks of hospital readmission, but with substantial investment of operational resources.^[7,8]^ With an eye towards informing economical health system approaches to reducing readmissions in our large urban health care system that serves a diverse community of patients with both public and private insurance, we set out to test the hypothesis that simply having a post-hospital visit with a primary care physician—without implementing costly wraparound interventions—is associated with lower readmission rates.

## 2. Methods

This is a retrospective cohort study of patients discharged from University of California, Los Angeles Health System (UCLA Health) hospitals. UCLA Health is a large academic integrated health system that includes a community hospital in Santa Monica, CA and a quaternary care hospital in West Lost Angeles, CA.

### 2.1. Patient population

The cohort includes patients 18 years and older who were discharged alive from inpatient hospital admissions or observation from January 1, 2019 to September 9, 2019 from 2 large academic hospitals with a PCP from the same academic center. We excluded discharges from psychiatry, labor and delivery, patients whose reason for admission was elective surgery, and patients who were discharged to an acute rehabilitation center or skilled nursing facility.

### 2.2. Data

#### 2.2.1. Measures.

A post-hospitalization PCP visit (independent variable) was defined as a visit with a primary care physician within 14 days of discharge, as defined by the transitional Care Management Services.^[9]^ Our outcome measure was readmission to a UCLA Health hospital for any condition within 30 days. Secondary analyses assessed if there was an association with reductions in hospital readmissions at 90 days and post-hospitalization PCP visits within 7 days of hospital discharge.

We measured the following sociodemographic characteristics that could be associated with having a PCP visit and/or readmission: age, sex, race and ethnicity, area deprivation index (ADI) score, LACE + index (Length of stay, Acute/emergent admission, Charlson comorbidity index score, Emergency department visits in the prior 6 months),^[10]^ and healthcare utilization measures, which included PCP visits in the last 36 months and hospital admissions in the prior 12 months. Variables that were included in the LACE + score were not used in order to not double count effects of variables (i.e., length of stay, comorbidities, and emergency department visits in the last 6 months). Race/ethnicity is self-reported and entered into the electronic health record at the time of profile creation. We also measured the length of stay on the index admission as a predictor of length of readmission. ADI, a measure based on data from the Health Resources and Services Administration that ranks neighborhoods by socioeconomic status disadvantage, was also assessed. ADI includes data on income, education, employment, and housing quality.^[11]^ ADI was stratified into deciles for our analysis.

#### 2.2.2. Analyses.

We performed bivariate analyses to measure the unadjusted associations between having a primary care physician visit within 14 or 7 days of hospital discharge and our primary and secondary outcomes of hospitalization within 30 and 90 days. We constructed multivariate logistic regression model to estimate the adjusted odds ratio of a patient experiencing a 30-day hospital readmission among patients with a PCP visit within 14 days of discharge (primary outcome). In secondary analyses, we constructed a multivariate logistic regression model to estimate the adjusted odds ratios of a patient experiencing a 90-day hospital readmission among patients with a PCP visit within 14 days of discharge as well as PCP visits withing 7 days of discharge. Characteristics that were associated with the readmission outcomes with a *P* value less than .2 in the bivariate analyses were included in the multivariate models. We performed statistical analyses using StataSE (StataCorp LLC., College Station, TX).

#### 2.2.3. Ethics.

This study was deemed exempt from review by the UCLA IRB, #19-001894.

## 3. Results

A total of 9236 patients met inclusion criteria. Patient characteristics are listed in Table 1. Of the study population, 35.6% (n = 3284) and 24.1% (n = 2224) of patients had a post-hospitalization PCP visit within 14 days and or 7 days, respectively. Overall, 1259 (13.6%) and 2153 (23.3%) of discharged patients were readmitted at 30 and 90 days, respectively.

**Table 1:** Patient characteristics.

		Primary care visit within 14 d		Primary care visit within 7 d	
	All patients	No	Yes	No	Yes
	N = 9236	N = 5952 (64.4%)	N = 3284 (35.6%)	N = 7012 (75.9%)	N = 2224 (24.1%)
Patient age (yrs)					
Mean (SD)	57.9 (20.1)	53.9 (19.7)	65.2 (18.7)	55.6 (19.9)	65.3 (18.7)
Median (IQR)	60.0 (39.0–74.0)	54.0 (36.0–70.0)	68.0 (52.0–79.0)	57.0 (37.0–71.0)	68.0 (52.0–79.0)
Sex					
Female	5513 (59.7%)	3739 (62.8%)	1774 (54.0%)	4346 (62.0%)	1167 (52.5%)
Hispanic					
Yes	1595 (17.3%)	998 (16.8%)	597 (18.2%)	1180 (16.8%)	415 (18.7%)
Missing	405	257	148	294	111
Race					
White	5467 (59.2%)	3487 (58.6%)	1980 (60.3%)	4138 (59.0%)	1329 (59.8%)
Asian	974 (10.6%)	675 (11.3%)	299 (9.1%)	763 (10.9%)	211 (9.5%)
Black	899 (9.7%)	544 (9.1%)	355 (10.8%)	654 (9.3%)	245 (11.0%)
Other/Unknown	1896 (20.5%)	1246 (20.9%)	650 (19.8%)	1457 (20.8%)	439 (19.7%)
ADI					
Mean (SD)	3.2 (2.5)	3.3 (2.5)	3.1 (2.5)	3.3 (2.5)	3.1 (2.5)
Median (IQR)	2 (1–5)	2 (1–4)	2 (1–5)	2 (1–4)	2 (1–5)
LACE + Score					
Mean (SD)	49.2 (24.8)	43.3 (26.4)	59.9 (17.0)	45.6 (25.9)	60.5 (16.3)
Median (IQR)	55 (29–71)	49 (14–68.5)	65 (52–72)	51 (18–70)	65 (53–72)
PCP visits prior to admission (last 36 mo)					
Mean (SD)	9.6 (10.0)	7.6 (8.1)	13.4 (11.8)	8.4 (8.9)	13.7 (11.9)
Median (IQR)	7 (3–13)	10 (5–18)	2 (1–5)	6 (3–11)	10 (6–18)
Admissions (last 12 mo)					
Mean (SD)	0.7 (1.2)	0.7 (1.2)	0.8 (1.2)	0.7 (1.2)	0.8 (1.2)
Median (IQR)	0 (0–1)	0 (0–1)	0 (0–1)	0 (0–1)	0 (0–1)

*ADI—Area Deprivation Index, a measure based on data from the Health Resources and Services Administration that ranks neighborhoods by socioeconomic status disadvantage. ADI includes data on income, education, employment, and housing quality. ADI was stratified into deciles, IQR = Interquartile Range.

**LACE + index (Length of stay, Acute/emergent admission, Charlson comorbidity index score, a measure of risk of death or nonelective 30-day readmission after hospital discharge).

The unadjusted bivariate analyses did not identify an association between patients with a post-hospitalization PCP visit (Table 2a). The unadjusted odds of hospital readmission at 90 days were 1.21, (95% CI 1.10–1.34) and 1.16, (95% CI 1.04–1.30) for patients with a PCP visit within 14 days and 7 days, respectively.

In our primary analysis, patients having a post-hospitalization PCP visit within 14 days of hospital discharge had a lower likelihood of being readmitted 30 days, adjusted odds ratio (aOR): 0.68 (95% CI 0.59–0.79; Table 2b). In our secondary analysis, patients having a post-hospitalization PCP visit within 7 days of hospital discharge had a lower likelihood of being readmitted 30 days, aOR: 0.76 (95% CI 0.66–0.89). For PCP visit within 14 and 7 days, the aOR of being readmitted within 90 days was 0.76 (95% CI 0.68–0.85) and 0.80 (95% CI 0.70–0.91), respectively. Patients who were male, older, had longer index admissions, were from a neighborhood with a worse area deprivation index score, had a higher LACE + score, and had more PCP visits in the last 36 months and hospital admissions in the last 12 months were at higher risk for hospital readmission.

## 4. Discussion

Among patients discharged from a large urban integrated academic health system in California, 14-day post-hospitalization PCP visits were associated with lower rates of hospital readmission at 30 days following hospital discharge. In secondary analyses, this association was also observed among 7-day post-hospitalization PCP visits, as well as 14- and 7-day post-hospitalization PCP visits for hospital readmissions within 90 days. Though causality cannot be proven in this observational study, this supports our hypotheses that PCP visits protect against hospital readmission, without other intensive interventions described in prior studies. The protective association that we found span was independent of sex, ADI, or age.

Hospital readmissions continue to remain a challenging and costly problem. There are many remaining barriers to accessing primary care following hospitalization including geographic accessibility, wait time, health care aversion, and insurance status.^[12,13]^

Our study adds to the large, sometimes conflicting, body of work on this topic by showing that: a primary care physician visit alone (not part of a costly labor-intensive program) is associated with lower rates of readmission and this is true in an integrated academic health system with diverse patients and payor bases.

Our study has limitations. Our study was observational in design; therefore, we cannot conclude there is a causal relationship for PCP visits and decreasing hospital readmissions. We were unable to capture post-hospital visits with a PCP nor hospital admissions outside of the UCLA Health network. We focused on patients who are part of our integrated health system with PCPs in our system, so this cannot be extended to patients who were not part of this system and may have had visits with PCPs and/or readmissions at other health systems. Our patient population is from a single healthcare system with high readmission rates and medically complex patients.

## 5. Conclusions

Timely post-hospitalization PCP visit after hospital discharge were associated with lower risk of hospital readmission at 30 and 90 days. However, many patients in our study cohort did not have a post-hospitalization PCP visit. This suggests that the single intervention of increasing rates of completed post-hospitalization PCP visits may be a powerful, low-intensity method to reduce hospital readmissions. More research is needed to determine whether this strategy can be successful in reducing hospital readmissions and to understand which patients benefit most from a post-hospitalization PCP visit.

**Table 2a:** Unadjusted analyses.

	Outcome = 30-d readmission				Outcome = 90-d readmission			
Independent variable	Odds ratio	95% CI		*P* value	Odds ratio	95% CI		*P* value
PCP visit within 14 d	0.99	0.87	1.12	.82	1.21	1.10	1.34	<.001
PCP visit within 7 d	0.99	0.86	1.14	.88	1.16	1.04	1.30	.009
Female	0.71	0.63	0.80	<.001	0.66	0.60	0.73	<.001
Age (yrs)	1.01	1.01	1.02	<.001	1.02	1.01	1.02	<.001
ADI score	1.03	1.00	1.05	.02	1.03	1.01	1.05	.009
LACE + score	1.03	1.03	1.04	<.001	1.04	1.04	1.04	<.001
PCP Visits in the last 36 mo	1.02	1.02	1.03	<.001	1.03	1.03	1.03	<.001
Hospital admissions in the last 12 mo	1.60	1.53	1.67	<.001	1.77	1.70	1.85	<.001

**Table 2b:** Multivariate analyses.

Multivariate models with outcome = 30-d readmission					Multivariate models with outcome = 90-d readmission				
Independent variable	Odds ratio	95% CI		*P* value	Independent variable	Odds ratio	95% CI		*P* value
PCP visit within 14 d	0.68	0.59	0.79	<.001	PCP visit within 14 d	0.76	0.68	0.85	<.001
Female	1.05	0.92	1.20	.45	Female	1.03	0.92	1.15	.60
Age (yrs)	0.99	0.99	1.00	.01	Age (yrs)	0.99	0.99	1.00	<.001
ADI score	1.02	0.99	1.04	.24	ADI score	1.02	1.00	1.04	.08
LACE + score	1.03	1.03	1.04	<.001	LACE + score	1.04	1.03	1.04	<.001
PCP Visits in the last 36 mo	1.00	1.00	1.01	.13	PCP visits in the last 36 mo	1.01	1.01	1.02	<.001
Hospital admissions in the last 12 mo	1.37	1.31	1.43	<.001	Hospital admissions in the last 12 mo	1.42	1.35	1.48	<.001
Independent variable	Odds Ratio	95% CI		*P* value	Independent variable	Odds ratio	95% CI		*P* value
PCP visit within 7 d	0.76	0.66	0.89	<.001	PCP visit within 7 d	0.80	0.70	0.91	<.001
Female	1.05	0.91	1.19	.52	Female	1.03	0.92	1.15	.66
Age (yrs)	0.99	0.99	1.00	.01	Age (yrs)	0.99	0.99	1.00	<.001
ADI score	1.02	0.99	1.04	.26	ADI score	1.02	1.00	1.04	.08
LACE + score	1.03	1.03	1.03	<.001	LACE + score	1.04	1.03	1.04	<.001
PCP visits in the last 36 mo	1.00	1.00	1.01	.31	PCP visits in the last 36 mo	1.01	1.00	1.02	<.001
Hospital admissions in the last 12 mo	1.37	1.31	1.43	<.001	Hospital admissions in the last 12 mo	1.42	1.36	1.48	<.001

*The primary outcome was adjusted by the other listed variables.

## Acknowledgments

We would like to acknowledge Lucia Y. Chen and Sitaram Vangala for assistance with statistical support and UCLA Health.

## Author contributions

**Conceptualization:** Noah Kojima, Andrea Sorensen, Chad Villaflores, Catherine Sarkisian.

**Data curation:** Marielle Bolano, Chad Villaflores.

**Formal analysis:** Noah Kojima.

**Investigation:** Marielle Bolano, Catherine Sarkisian.

**Methodology:** Chad Villaflores, Catherine Sarkisian.

**Project administration:** Chad Villaflores.

**Resources:** Catherine Sarkisian.

**Supervision:** Andrea Sorensen, Chad Villaflores, Daniel Croymans, Eve M. Glazier, Catherine Sarkisian.

**Validation:** Eve M. Glazier, Catherine Sarkisian.

**Visualization:** Catherine Sarkisian.

**Writing – original draft:** Noah Kojima.

**Writing – review & editing:** Noah Kojima, Marielle Bolano, Andrea Sorensen, Daniel Croymans, Eve M. Glazier, Catherine Sarkisian.
